# Differential diagnosis of brain lesions in a metastatic endometrial carcinosarcoma patient

**DOI:** 10.3332/ecancer.2021.1182

**Published:** 2021-02-09

**Authors:** Asrie Arsad, Clement Yong, Desmond Boon Seng Teo

**Affiliations:** 1 Division of Advanced Internal Medicine, Department of Medicine, National University Hospital, National University Health System, 1E Kent Ridge Road, Singapore 119228, Singapore; 2 FAST Programme, Alexandra Hospital, National University Health System, 378 Alexandra Road, Singapore 159964, Singapore; 3 Department of Diagnostic Imaging, National University Hospital, National University Health System, 1E Kent Ridge Road, Singapore 119228, Singapore; 4 Instructor, Department of Medicine, Yong Loo Lin School of Medicine, National University of Singapore, Singapore

**Keywords:** diagnosis, differential diagnosis, brain neoplasms, toxoplasmosis

## Abstract

The differential diagnosis of ring-enhancing brain lesions in a patient with metastatic malignancy may initially seem straightforward, and easily attributed to brain metastases. On rare occasions, the physician needs to avoid anchoring bias by re-evaluating the entire clinical context in which these ring-enhancing brain lesions are found. We report a case of cerebral toxoplasmosis mimicking brain metastases in a patient with metastatic cancer and without a prior history of human immunodeficiency virus. A 65-year-old lady with a recently detected relapse of her endometrial carcinosarcoma presented with a 2-week history of fever with no localising symptoms or signs of infection. The initial investigations were unremarkable. She had daily fever despite empirical broad-spectrum antibiotics. A positron emission tomography-computed tomography (PET-CT) was performed to evaluate the pyrexia of unknown origin, which showed metastatic deposits in the pelvis. A magnetic resonance imaging (MRI) of the brain was subsequently performed due to fluctuating mentation, which reported metastatic disease to the brain. Her pyrexia of unknown origin was attributed to malignancy-related fever. The medical oncologist was cautious about starting systemic treatment because the PET-CT had FDG-avid diffuse ground glass opacities in both lung fields, and requested for a bronchoscopic evaluation, which returned positive for *Pneumocystis jirovecii*. In light of this new finding, a multi-disciplinary discussion and a review of the brain MRI were undertaken, during which it was concluded that the likelihood of cerebral toxoplasmosis was much higher than brain metastases. She was treated with high dose trimethoprim-sulfamethoxazole for both *P. jirovecii* pneumonia and cerebral toxoplasmosis, with clinical and radiological improvement. This case highlights the importance of (a) clinical input in interpreting imaging findings, (b) entertaining the possibility of multiple concurrent pathologies explaining a patient’s symptoms, (c) being open to alternate diagnoses when new information surfaces even though the current working diagnosis is the most plausible and (d) multi-disciplinary communication when faced with diagnostic difficulty.

## Presentation

A 65-year-old lady presented with a 2-week history of fever, 38° to 40°, chills and rigors. She had no localising symptoms of infection, and there was no recent sick contact or travel. She has a history of poorly controlled type 2 diabetes mellitus (HbA1c 10%–15%), ischaemic heart disease and underwent total hysterectomy and bilateral salpingo-oophorectomy followed by vaginal brachytherapy for Stage 1 endometrial carcinosarcoma a year ago. She was found to have tumour recurrence in her pelvis during a surveillance computed tomography (CT) of the pelvis 2 months prior to admission, and was scheduled to undergo pelvic radiotherapy, but was postponed due to her fever.

## Assessment

On examination, she was febrile at 39°, pale and lethargic. There were no localising signs of infection. Abdominal examination did not reveal any masses. She did not have leucocytosis, but had microcytic anaemia of 5.6 g/dL (normal 11.4–14.7). Chest radiograph, urine microscopy and cultures, liver function test, C-reactive protein, procalcitonin and repeated blood cultures were unremarkable.

**Figure 1.**
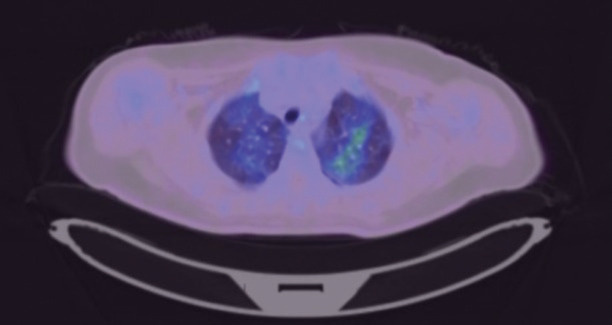
PET-CT showing FDG-avid diffuse GGOs in both lung fields.

She had daily fever despite empirical broad-spectrum antibiotics. A positron emission tomography-CT (PET-CT) was performed to evaluate the pyrexia of unknown origin. The PET-CT showed a hypermetabolic soft tissue mass in the left pelvis and sigmoid colon, suspicious for metastatic deposits. Incidentally, the PET-CT picked up FDG-avid diffuse ground glass opacities (GGOs) in both lung fields (Figure 1), which were not present in the contrast-enhanced thorax CT performed 3 months ago.

Her pyrexia of unknown origin was attributed to malignancy, and Medical Oncology consult was sought to discuss about systemic treatment for her metastatic endometrial carcinosarcoma. The oncologist was cautious about starting systemic treatment in light of the FDG-avid bilateral diffuse GGOs, and requested for a consult with Respiratory Medicine, whom requested for induced sputum for tuberculosis testing, which was negative. The Respiratory physician opined that the FDG-avid bilateral diffuse GGOs could be related to metastatic malignancy.

She continued to have daily fever and subsequently became oxygen dependent. The oncologists were worried about an undiagnosed lung infection, which could worsen with systemic treatment, and thus requested for a bronchoscopic evaluation.

**Figure 2.**
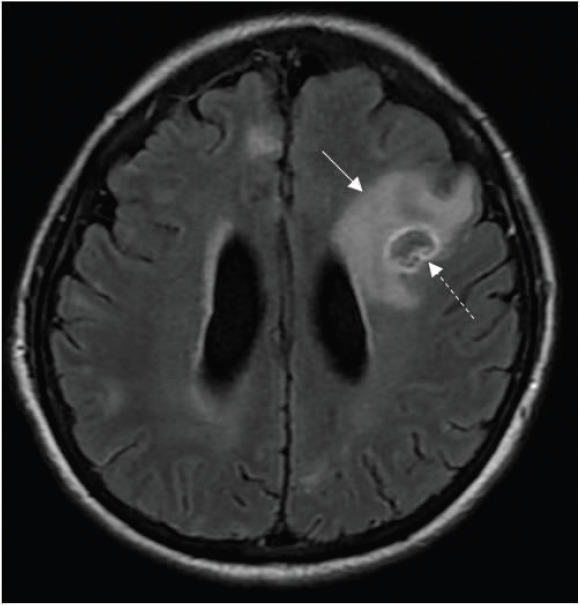
Axial T2-FLAIR post contrast-enhanced image showing a ring-enhancing lesion in the left frontal lobe with significant perilesional oedema (solid arrow). An eccentric focus of mural enhancement is seen (dashed arrow).

**Figure 3.**
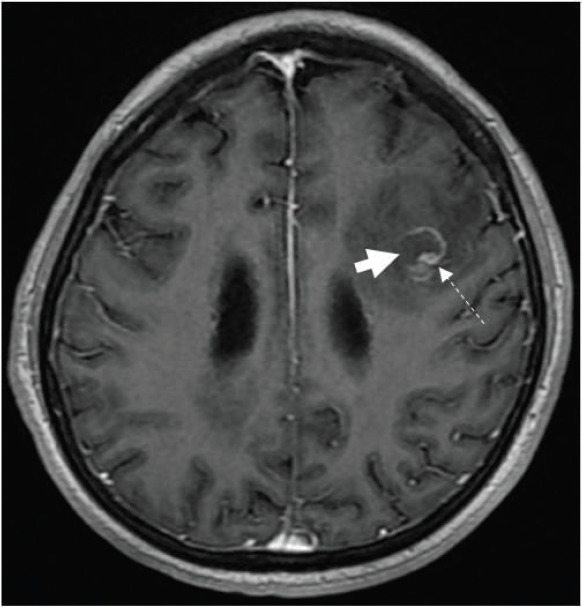
Axial T1-weighted post contrast-enhanced image showing an incomplete ring-enhancing lesion (short arrow) in the left frontal lobe. An eccentric focus of mural enhancement is seen (dashed arrow).

**Figure 4.**
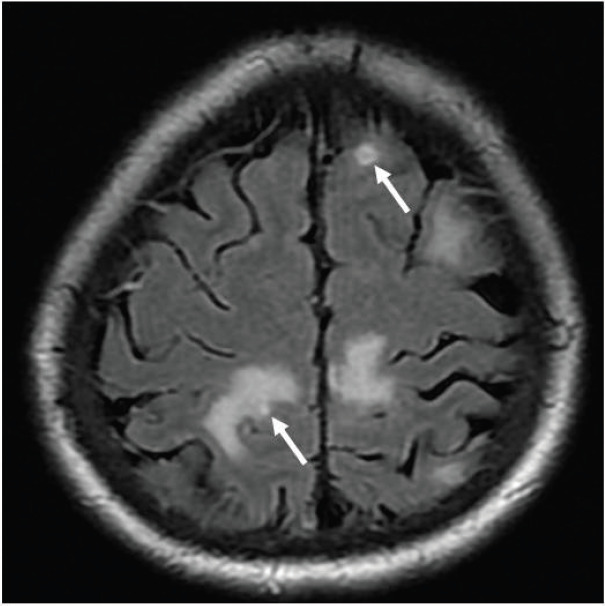
Axial T2-FLAIR post contrast-enhanced image showing other lesions within the bilateral grey white junction, some with focal enhancement (thick arrows).

While arranging for bronchoscopy, her mentation fluctuated, with occasional disorientation. In light of the working diagnosis of malignancy-related fever, a contrast-enhanced magnetic resonance imaging (MRI) of the brain was performed to exclude brain metastases, which reported multiple ring-enhancing lesions in the cerebrum and cerebellum consistent with metastatic disease (Figures 2–4).

She underwent bronchoscopy soon after the brain MRI, and the bronchoalveolar lavage (BAL) was positive for *Pneumocystis jirovecii* (*P. jirovecii)*, which was initially thought to be secondary to an immunocompromised state from her malignancy and poorly controlled diabetes. She was also screened for human immunodeficiency virus (HIV) in view of the BAL results.

## Diagnosis

Radiation Oncology was sought to discuss radiotherapy for symptomatic brain metastases. Upon review of her brain MRI, they were cautious about recommending whole brain radiation therapy in light of the new diagnosis of *P. jirovecii* pneumonia and pending HIV test. A multi-disciplinary meeting among Infectious Diseases, Radiation Oncology, Medical Oncology and Radiology was conducted to discuss the differential diagnoses of her multiple ring-enhancing brain lesions, and it was concluded that the likelihood of cerebral toxoplasmosis was much higher than brain metastases.

Anti-toxoplasma IgG antibodies returned positive 3 days later, while the IgM antibodies were absent.

## Management

She was started on high dose trimethoprim-sulfamethoxazole for both *P. jirovecii* pneumonia and cerebral toxoplasmosis.

Her HIV test returned positive 2 days later, and her CD4 count was 28 cells/μL (normal 500–1,500 μL). She had protected sex with two men previously, but had been sexually inactive for 20 years. She had neither blood product transfusion nor intravenous drug abuse in the past.

**Figure 5.**
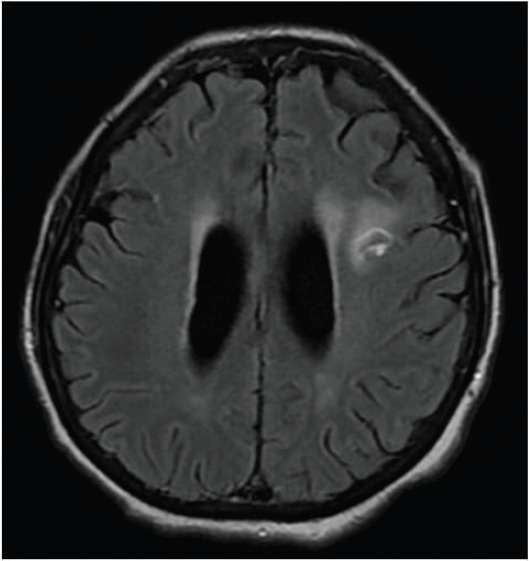
Reduction in size of the ring-enhancing lesion and resolution of oedema (compared to Figure 2). A follow-up MRI performed after 3 weeks of treatment showed interval reduction in size of the ring-enhancing lesion and resolution of the oedema (as compared to Figure 2). The other smaller lesions were also less well appreciated on this study.

Her fever resolved and her mentation improved. Repeat brain MRI after 3 weeks of treatment showed generalised decrease in the size of ring-enhancing lesions, with dramatic decrease in the associated oedema and mass effect (Figure 5).

## Discussion

The evaluation of pyrexia of unknown origin can be challenging, and requires systematic evaluation with repeated history taking, physical examination and targeted investigations [1]. After excluding common infections with failure to respond to broad-spectrum antibiotics, malignancy-related fever was considered the next likely culprit, especially with her recently detected pelvic tumour recurrence. The brain MRI that was performed due to fluctuating mentation reported what initially seemed to be consistent with this clinical suspicion.

While FDG-avid bilateral diffuse GGOs are a non-specific finding that has many differentials, including *P. jirovecii*, it is important to rule out infections prior to systemic cancer therapy due to concerns of immunosuppression, and hence the bronchoscopic evaluation.

*Pneumocystis jirovecii* is an AIDS-defining condition, and its diagnosis should prompt a thorough evaluation of other opportunistic infections and malignancies. It commonly occurs when the CD4 count is less than 200 cells/μL [2].

The patient’s HIV test result and CD4 count were not available prior to the diagnosis of cerebral toxoplasmosis. While a positive HIV test and low CD4 count may lend greater weight to the diagnosis of cerebral toxoplasmosis and cerebral lymphoma over brain metastases from endometrial carcinosarcoma, there were several subtle radiological features which favoured cerebral toxoplasmosis over brain metastases and lymphoma [3, 4]. These were:

- Ring-enhancing lesions had eccentric nodular enhancement (Figures 2 and 3).
- Involvement of basal ganglia and corticomedullary regions (Figures 4).
- Presence of oedema with mass effect (Figure 2).

In the setting of HIV, the differential diagnoses of ring-enhancing lesions in the brain are extensive, depending on the CD4 count. At CD4 counts of less than 200 cells/μL, the most common differential diagnoses are toxoplasmosis, lymphoma, abscesses, metastases, tuberculosis and fungal infections. In addition, multiple etiologies can coexist in immunosuppressed patients [5].

The main differential diagnosis for cerebral toxoplasmosis with regard to imaging, and in particular for this patient with a CD4 count of 28 cells/μL, is central nervous system (CNS) lymphoma [3, 6]. CNS lymphoma tends to be locally infiltrative, is usually located in a periventricular or periependymal location, and tends to be solitary, while toxoplasma lesions tend to be multiple and located in the basal ganglia, thalamus and corticomedullary regions. Less than 10% of lymphomas involve the posterior fossa; thus, posterior fossa lesions are more likely to be due to an alternative process such as infection [6]. Toxoplasma lesions may show peripheral hyperintensity on T1-weighted MRI, but this is less commonly seen in lymphoma. The eccentric target sign on contrast-enhanced T1-weighted images, which exhibits a small enhancing nodule along the wall of the enhancing ring, is highly specific for cerebral toxoplasmosis, but is seen in less than 30% of cases [4]. In addition to brain MRI, PET-CT can further help distinguish cerebral toxoplasmosis from CNS lymphoma, as the standardised uptake is much higher for CNS lymphoma than toxoplasmosis [4].

The context in which the ring-enhancing lesions are found is probably the most important clue, as subtle features on radiology may not be sufficient to pinpoint the exact diagnosis. Even when an obvious cause for cerebral ring-enhancing lesions is apparent (e.g. tumour recurrence in this patient), a thorough review of the patient’s clinical progression and investigations for features which do not fit into the initial impression may lead to the correct alternative diagnosis.

## Conclusion

The physician should entertain the possibility of multiple concurrent pathologies explaining a patient’s symptoms, rather than trying to fit everything into a single diagnosis. Being open to alternate diagnoses when new information surfaces and good multi-disciplinary communication when faced with diagnostic difficulty are essential because for the same imaging finding, relevant clinical information can significantly change the post-test probability of a particular condition.

## Conflicts of interest

The authors declare that they have no conflict of interest.

## Funding

No funding was required for this manuscript.

## Authors’ contributions

All authors had access to the data and a role in writing the manuscript.

Asrie Arsad: conceptualisation, writing—original draft, writing—review and editing.

Clement Yong: resources, writing—review and editing.

Desmond Boon Seng Teo: writing—review and editing, supervision, project administration.
